# Editorial: Multidisciplinary management of urological malignancies in the era of precision medicine: integration of advances in technology and cancer care

**DOI:** 10.3389/fruro.2024.1518410

**Published:** 2025-01-08

**Authors:** Lilia Bardoscia, Beatrice Detti, Angela Sardaro

**Affiliations:** ^1^ Radiation Oncology Unit, S. Luca Hospital, Healthcare Company Tuscany Nord Ovest, Lucca, Italy; ^2^ Radiotherapy Unit Prato, Usl Centro Toscana, Presidio Villa Fiorita, Prato, Italy; ^3^ Department of Radiation Therapy, "Vito Fazzi" Hospital, Lecce, Italy; ^4^ University of Bari “Aldo Moro”, Bari, Italy

**Keywords:** urology malignancies, stereotactic body radiotherapy, robotic surgery, multidisciplinary tumor board, next generation imaging

Urologic cancer burden has globally increased amid population growth and aging (1). The routine use of advanced imaging modalities, such as multiparametric magnetic resonance (MRI) and disease-specific tracers positron emission tomography in combination with computed tomography or MRI has improved the early detection of these tumors, local recurrence or distant progression (2, 3). A large amount of morphological, functional and molecular data can be obtained from next-generation imaging and guide a reliable identification of insights on tumor heterogeneity, thus the implementation of diagnosis and targeted, personalized treatments (4–6).

The presented Research Topic aimed to create a multidisciplinary collector of scientific evidence that strengthen communication among the disciplines involved in a Uro-oncology tumor board (Urology, Radiation Oncology, Clinical Oncology, Radiology, Nuclear Medicine, Pathology, Molecular Biology), exploring technological advances in the field of prostate cancer (PCa), urothelial and renal tumors, and their impact on cancer care, patients attitudes or preferences.

The development of robot-assisted surgery has allowed numerous potential benefits to patients, reduced hospital stay, minor risk of infection and postoperative complications than conventional surgery. Urologists have been pioneers, innovative and flexible robotic systems allowed more efficient, precise and accurate surgical procedures than in the past (7, 8). Nevertheless, the diagnostic possibility of depicting metabolic activity, receptor expression, oxygenation or cellular density of healthy tissues and the tumor mass, together with technological advances in radiotherapy planning and delivery techniques have made it possible to perform effective radiation treatments with a relatively low toxicity profile, in the primary tumor and selected cases of recurrent/metastatic setting (9–15). Lancia et al. provided an interesting overview on personalization of the use of radiotherapy based on biological information by functional imaging integrated into the linear accelerator for both primary tumor and metastases-directed therapy of metastatic PCa. The advent of volumetric multiple arc and rotational/helical intensity-modulated radiotherapy allowed steep dose gradients, spatially non-uniform dose distributions with improved sparing of the surrounding normal tissues (16). Stereotactic ablative body radiotherapy/radiosurgery schedules have been readily incorporated into the oncology routine clinical practice, as a safe and cost-effective part of multimodal, patient-tailored, therapeutic strategies enabling high doses delivered in only three to five fractions with curative intent (17, 18).

The growing evidence of improved disease control and survival across the recent advances in the diagnosis and treatment of prostate, urothelial and renal cancers, even in the setting of oligorecurrent/oligometastatic disease, requires focusing on patients long-term treatment-related quality of life (QoL), as well. In this Research Topic, Qian et al. introduced the application of the gratitude extension construction theory-nursing program to a cohort of patients surgically treated for bladder cancer. Clinicians and allied health professionals often tend to place different utilities, higher scores to health states than patients undergoing treatment procedures (19). The use of validated, self-administered questionnaires helps understand the real patients perception of their clinical condition and fear of cancer recurrence, while high gratitude allow individuals to experience positive emotions, making them be aware, collaborative and confident in the long-term success of the prescribed cancer treatment.

The current trend towards the hyperspecialized combination of diagnostics and therapeutics outlines the lack of reliable predictors of advanced disease and treatment response of primary and metastatic urology malignancies, to improve health counselling. Prostate-specific antigen (PSA) is still the only validated biomarker in Uro-oncology, the best known and widespread tool for early detection of PCa, although screening for PCa is a controversial topic given the high risk of identifying insignificant cancer and overtreatment while preventing disease-specific mortality (20). Hsieh et al. reported their experience of health screening in a Taiwanese men rural community below, highlighting the opportunity for a careful interpretation of high blood PSA levels within the context of patient age and lifestyle, since it might also underlie non-neoplastic, but equally life-threatening diseases related to unhealthy lifestyle habits like PCa (i.e. cardiometabolic syndromes).

In the artificial intelligence era, both clinical and molecular data extraction is crucial to optimize new, customized treatment modalities targeting specific markers of tumor aggressiveness and clonal evolution. For instance, Colosini and colleagues investigated the contribution of circulating cell-free DNA gene sequencing underlying a true oligometastatic PCa state, with slower and more favorable evolution than polymetastatic disease (21). Similarly, tumor-cell derived microRNAs delivered by exosomes have shown to have a role in the tumorigenesis promotion, whose quantification or expression panel has been demonstrated to affect cell proliferation, invasiveness and removal capability, and predict survival in renal cell cancer cases (22, 23). Liu et al. described bladder cancer organoid models as a potential guide for treatment selection. These are *in-vitro*, three-dimensional tumor models faithfully reproducing histological architecture and mutational burden of the parental tumor (24). Based on a differential molecular or phenotype cell characterization, or drug sensitivity tests, urothelial cancer organoids appear as a promising way to submit patients to conventional (neoadjuvant chemotherapy followed by cystectomy) or alternative treatments (bladder-sparing (chemo)radiation, immunotherapy or antibody-drugs conjugates) (25), in expectation of poor response to the standard of care or recurrent tumors unfit for radical surgery.

The integration of histopathology data with radiomic and molecular features, routine clinicopathological and common risk-stratification parameters, patient-completed tools combined with physician grading of symptoms, possibly through automated deep learning workflows, is the future key for a multidimensional, comprehensive understanding of tumor behavior driving an accurately defined anticancer treatment intensification or deintensification that is certainly harbinger of disease control. Synergy within a multidisciplinary Uro-oncology team is also essential to adequately frame clinic findings and patients perception, therefore ensure quality, multimodal cancer care. Actually, the selection of patients for cancer treatment is based on patients characteristics (age, performance status, comorbidity) and tumor mass-related factors like tumor size and histological subtype, while the use of information on tumor biology is still limited. The real-time molecular characterization of tumor vulnerability, monitoring of therapeutic responses, and tracking minimal residual disease represents the beginning of a new era of precision medicine, where not only further individualization of radiation dose, surgical or systemic therapy prescription is possible, but also the goals of concrete reduced toxicity and improved QoL of long-term cancer survivors become definitely achievable.
